# Morphological Variability of Sphenoid Sinus Pneumatization and Its Impact on Adjacent Neurovascular Structures

**DOI:** 10.3390/diagnostics16050809

**Published:** 2026-03-09

**Authors:** Panagiotis Papadopoulos-Manolarakis, George Triantafyllou, Christos Georgalas, Ioannis Paschopoulos, George Stranjalis, Maria Piagkou

**Affiliations:** 1 Department of Anatomy, School of Medicine, Faculty of Health Sciences, National and Kapodistrian University of Athens, 11527 Athens, Greece; p.papado89@gmail.com (P.P.-M.); georgerose406@gmail.com (G.T.); johnpascho@gmail.com (I.P.); 2 Department of Neurosurgery, General Hospital of Nikaia-Piraeus, 18454 Athens, Greece; 3 Department of Otorhinolaryngology Head and Neck Surgery, University of Nicosia Medical School, 2408 Nicosia, Cyprus; cgeorgalas@gmail.com; 4 Endoscopic Skull Base Centre Athens, Hygeia Hospital, 15123 Athens, Greece; 5 Department of Neurosurgery, Evangelismos Hospital, School of Medicine, Faculty of Health Sciences, National and Kapodistrian University of Athens, 10676 Athens, Greece; gstranjalis@med.uoa.gr

**Keywords:** sphenoid sinus, pneumatization, internal carotid artery, optic nerve, Vidian nerve, maxillary nerve, variation, endoscopic transsphenoidal surgery

## Abstract

**Background/Objectives**: The sphenoid sinus (SS) exhibits marked morphological variability, influencing the relationship of critical neurovascular skull base structures. This study aimed to characterize sphenoid sinus pneumatization (SSP) patterns and assess their impact on the course of the internal carotid artery (ICA), optic nerve (ON), Vidian nerve (VN), and maxillary nerve (MN) within a Greek adult population. **Methods**: A retrospective analysis of 253 adult skull base computed tomography (CT) scans was performed. The degree and direction of SSP were classified according to established radiological criteria. Anterior, lateral, and posterior extensions were evaluated. The course of adjacent neurovascular structures was categorized as typical, protruding, or dehiscent. Associations between pneumatization types and neurovascular variants were analyzed. **Results**: The sellar complete type was the predominant SS pattern (63.2%), followed by sellar incomplete (27.7%) and presellar (8.7%) types; agenesis was rare (0.4%). Posterior (63.6%) and lateral (46.6%) extensions were most common. Lateral and posterior pneumatization significantly correlated with protrusion and/or dehiscence of adjacent neurovascular structures, particularly the ICA, ON, and VN. LW extension was strongly associated with ON protrusion (96%), while PP and full-lateral extensions correlated with VN protrusion (56.1% and 79.9%, respectively). No significant sex- or side-related differences were identified. **Conclusions**: SSP demonstrates extensive morphological variability that significantly affects the anatomical course and osseous coverage of neighboring neurovascular structures. Comprehensive preoperative CT evaluation of SS anatomy is essential for planning endoscopic transsphenoidal and extended skull base procedures to minimize the risk of neurovascular injury.

## 1. Introduction

The sphenoid sinus (SS) is a centrally located, developmentally intricate cavity that pneumatizes the sphenoid bone, variably extending into the lesser and greater wings (LWs and GWs), the clivus, and the pterygoid processes (PPs). Owing to its deep position within the skull base, it lies in proximity to several critical neurovascular structures, including the internal carotid arteries (ICAs), optic nerves (ONs), cranial nerves within the cavernous sinus, and the Vidian and maxillary nerves (VN and MN) [1,2,3,4,5,6,7,8,9,10].

The adult morphology of the SS reflects the complex embryological ossification, resulting in considerable variability that significantly influences surgical corridors and associated risk profiles [11]. The formation of the sphenoid bone itself begins around the third week of gestation, influenced by the notochord and mesenchymal tissues. By approximately day 40, four distinct cartilaginous primordia—the orbitosphenoid, alisphenoid, presphenoid, and postsphenoid—develop from separate chondrogenesis centers [11]. The SS initiates its formation from epithelial outpouchings of the nasal cavity. This process of sinus formation is vital for the later development of SS pneumatization and its physical relationship with adjacent sinuses [11]. The bone surrounding the sinus develops through a dual process of endochondral and intramembranous ossification [11]. As these regions ossify and fuse during the third trimester and into adolescence, the SS expands into these bony territories [11].

Historically, Hammer and Radberg [12] proposed the seminal classification of the SS into conchal, presellar, and sellar types, based on the relationship between pneumatization and the sella. This framework remains the cornerstone for transsphenoidal approaches to the sellar region. Later refinements introduced directional classifications that considered the extent and trajectory of pneumatization beyond the sella [12]. Wang et al. [13] further systematized these extensions—anterior, lateral (involving LW, GW, and PP), and posterior (clival)—into a comprehensive anatomical map of sinus “windows” to the skull base, emphasizing developmental progression and its surgical implications. Subsequent radiologic studies [14] and operative classifications [15] expanded on this model, highlighting that complex, combined patterns of pneumatization are more common than isolated variants.

From a surgical perspective, the SS has evolved from being considered a simple conduit to the sella to a modular gateway providing access to multiple skull base compartments. The development of microsurgical mapping of the sellar and parasellar regions laid the groundwork for endoscopic and extended endonasal techniques, broadening indications to include the planum sphenoidale, suprasellar, clival, and transpterygoid regions [16,17]. Despite these advances, specific morphologic variants—such as minimal or absent pneumatization, or the presence of kissing or ectatic carotid arteries—continue to favor transcranial approaches. Thus, SS anatomy, and specifically its pneumatization pattern, remains a key determinant of both surgical feasibility and safety [13,16,17].

Against this background, the present study aimed to perform a detailed radiologic analysis of non-pathological skull bases to characterize patterns of sphenoid sinus pneumatization (SSP) and to evaluate their relationship with adjacent neurovascular structures. Ultimately, the purpose is to investigate the potential association of specific pneumatization patterns with neurovascular structures and to characterize them statistically. The surgical significance of these results will be further discussed, especially for the preoperative assessment within the Greek adult population for which there is limited literature of such findings.

## 2. Materials and Methods

### 2.1. Sample Demographics

We retrospectively reviewed 253 adult skull bases to assess SS morphology and its relationships to adjacent neurovascular structures. The cohort comprised 129 males and 124 females (mean age 51.73 ± 15.02 years; range 18–90 years). Studies were randomly selected from the imaging archive of the General Hospital of Nikaia–Piraeus following institutional review board approval (protocol 56485; 13 November 2024). Of 300 initially screened CT datasets, 47 were excluded due to suboptimal image quality or pathologies that could distort local anatomy (such as, active SS pathology—acute or chronic sphenoiditis or neoplasms, space-occupying lesions—intracranial tumors, and prior sinonasal surgery), yielding the final analytical sample of 253.

### 2.2. Radiological Protocol

Each patient underwent a high-resolution sinus CT and a matched CT angiography (CTA) to correlate SS morphology with the course of adjacent vascular structures. Imaging was performed on a 128-slice helical low-dose scanner (SOMATOM go.Top, Siemens, Munich, Germany) with 0.4–0.8 mm collimation, 0.5 s rotation time, 0.8 pitch factor and 0.4 mm slice increment. The bone window was set at 2500 HU width and 480 HU level. Patients were positioned supine with the head in neutral alignment. Following the sinus CT, 60 mL of iodinated contrast (30% iodine) was injected at 4.0–4.5 mL/s for CTA acquisition. Images were analyzed in Horos (v3.3.6, Horos Project, New York, NY, USA) using multiplanar reconstructions (axial, coronal, sagittal) and three-dimensional (3D) volume renderings.

### 2.3. Methodology

**Figure 1:**
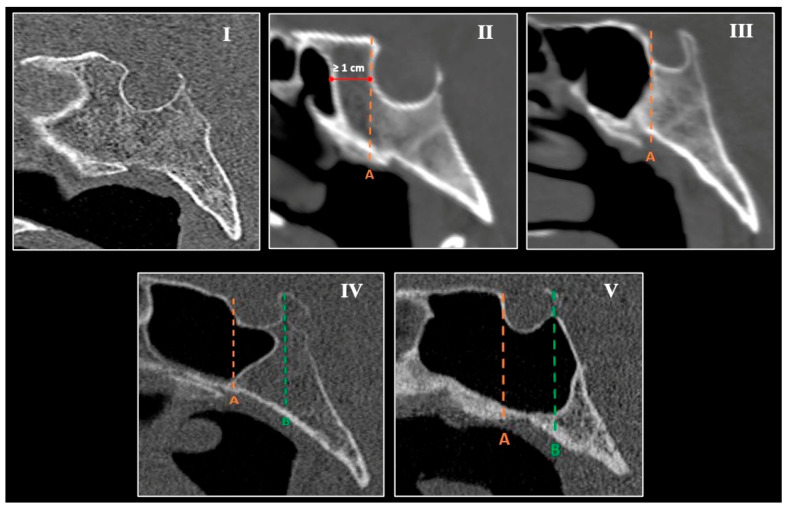
Main types of sphenoid sinus pneumatization (SSP) on midsagittal computed tomography (CT) images. Representative midsagittal CT sections demonstrating the five principal morphologic variants of the sphenoid sinus (SS) based on the relationship of pneumatization to the sella: (**I**) agenesis—complete absence of sinus cavity; (**II**) conchal type—minimal pneumatization not extending to the anterior wall of the sella (≥1 cm anterior to it); this case derived from the excluded population and it was not observed in the current sample; (**III**) presellar type—pneumatization reaches but does not pass the anterior wall of the sella; (**IV**) sellar incomplete type—pneumatization extends to the posterior clinoid process, involving both the anterior wall and sellar floor; (**V**) sellar complete type—pneumatization extends beyond the posterior wall of the sella into the basilar portion of the occipital bone. Dotted orange line (A) = vertical coronal plane through the anterior sellar wall; dotted green line (B) = plane through the posterior sellar wall.

Main SS types. On midsagittal images, SSP was classified according to Hiremath et al. [14] (Figure 1):

1. Agenesis—absence of the SS;
2. Conchal—slight pneumatization; sinus does not reach the anterior sellar wall and terminates ≥10 mm anterior to it;
3. Presellar—pneumatization limited to the vertical plane of the anterior sellar wall;
4. Sellar incomplete—pneumatization reaches the vertical plane of the posterior clinoid process, involving the anterior wall and sellar floor;
5. Sellar complete—pneumatization extends beyond the sella toward the basilar occiput.

**Figure 2:**
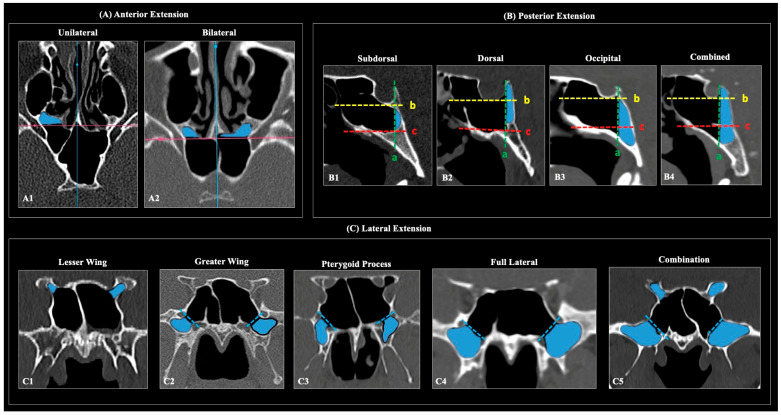
Patterns of sphenoid sinus pneumatization (SSP) on computed tomography (CT) according to anterior, posterior, and lateral extensions (blue colored). (**A**) Anterior extension: unilateral (**A1**) and bilateral (**A2**) pneumatization extending anteriorly beyond the anterior wall of the sphenoid sinus—SS (pink horizontal line). (**B**) Posterior (clival) extension: (**B1**) subdorsal—pneumatization extends posteriorly beyond the posterior wall of the sella (a, dotted green line) but remains between the line along the sellar floor (b, dotted yellow line) and the horizontal plane through the superior margins of the paired Vidian canals (c, dotted red line); (**B2**) dorsal—pneumatization extends above line (b); (**B3**) occipital—pneumatization extends below line (c); (**B4**) combined—pneumatization involves the entire clivus, extending both above (b) and below (c). Blue contours delineate the extent of pneumatization in each variant. (**C**) Lateral extension: pneumatization patterns involving (**C1**) the lesser wing—LW, (**C2**) greater wing—GW, (**C3**) pterygoid process—PP, (**C4**) full lateral—FL (GW+ PP), (**C5**) combined extension types.

Directional extensions. Extension patterns were assessed separately following Wang et al. [13] (Figure 2):

- Anterior extension (axial): pneumatization extending anteriorly beyond a line drawn along the sinus side of the sphenoid crest.
- Posterior (clival) extension (sagittal): subtypes—subdorsal, dorsal, occipital, and combined (dorsal + occipital)—defined relative to the posterior sellar wall, sellar floor line, and the horizontal plane through the superior margins of the paired Vidian canals.
- Lateral extension (coronal): presence of pneumatization lateral to the SS categorized as LW, GW, PP, full-lateral (GW + PP), and combinations thereof.

**Figure 3:**
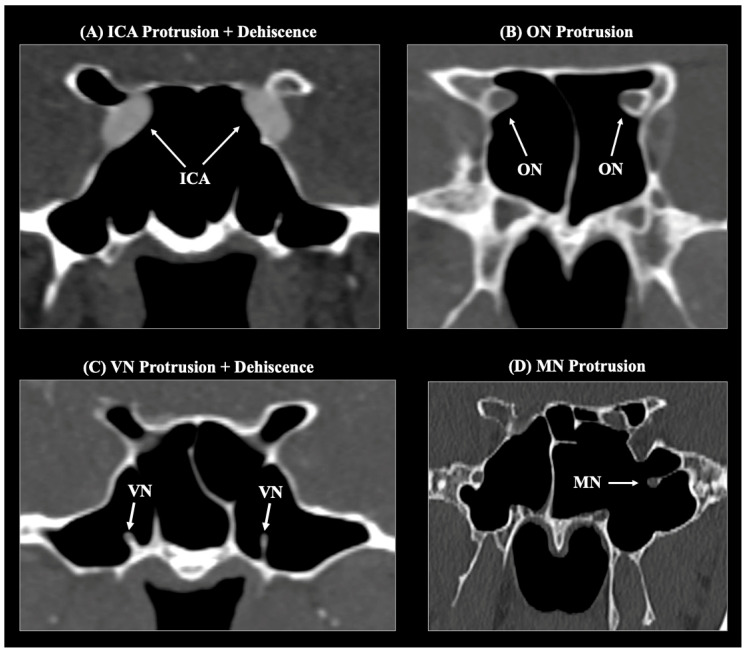
Representative computed tomography (CT) images demonstrating protrusion and dehiscence of key neurovascular structures into the sphenoid sinus (SS). (**A**) Internal carotid artery (ICA): bilateral protrusion with bony dehiscence. (**B**) Optic nerve (ON): bilateral protrusion into the superolateral wall of the SS. (**C**) Vidian nerve (VN): bilateral protrusion and dehiscence within a pneumatized SS showing combined lateral extension. (**D**) Maxillary nerve (MN): protrusion along the lateral wall of a full-lateral pneumatized SS. White arrows indicate the corresponding neurovascular structures.

The ICA, ON, Vidian nerve (VN), and maxillary nerve (MN/V2) were evaluated for their course relative to the SS and classified as typical (no protrusion) or protruding into the sinus, each with/without bony dehiscence (Figure 3).

**Figure 4:**
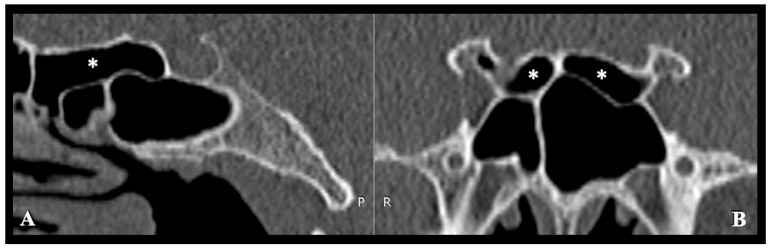
Computed tomography (CT) images demonstrate the presence and anatomical relationships of Onodi cells. (**A**) Sagittal CT image showing a unilateral Onodi cell (single asterisks, *) extending posteriorly and superolaterally to the sphenoid sinus (SS). (**B**) Coronal CT image revealing bilateral Onodi cells (single asterisks, *) positioned superior to the SS and closely related to the optic nerve canal. The Onodi cell, also known as the sphenoethmoidal cell, represents the most posterior ethmoidal air cell, which may extend into the sphenoid bone and create a potential risk for optic nerve or internal carotid artery exposure during endoscopic sinus or skull base procedures.

Protrusion was defined as >50% of the structure/canal circumference exposed to air within the SS. Dehiscence was defined as the partial or complete absence of the bony lamella over the structure along its intrasinus course [18,19]. The presence of Onodi (sphenoethmoidal) cells, are posterior ethmoid air cells located close to critical anatomical structures (ON and ICA), and their relationships to the ON and ICA were recorded (Anusha et al. [20]; Figure 4).

Each dataset was reviewed by three investigators (a neurosurgeon, a skull base otorhinolaryngologist, and an anatomist) who reached consensus interpretations to minimize interobserver bias. Interrater reliability was analyzed using Spearman’s r, with a pre-specified acceptable threshold of r ≥ 0.70 and α = 0.05 for statistical significance. Discrepant cases underwent additional review; a fourth investigator was consulted when necessary to achieve consensus.

### 2.4. Statistical Analysis

Statistical analyses were performed in IBM SPSS Statistics for macOS (v29, IBM Corp., Armonk, NY, USA). Graphs were generated in RStudio (v4.3.2; ggplot2). Nominal unpaired data were compared using the Chi-square test; paired nominal data were analyzed with McNemar’s test. Two-sided *p* < 0.05 was considered statistically significant. The predictive risk of neurovascular protrusion or dehiscence associated with specific pneumatization patterns was quantified using logistic regression to calculate Odds Ratios (ORs) and 95% Confidence Intervals (CIs).

## 3. Results

### 3.1. Sphenoid Sinus Pneumatization—Main Types

Among the 253 patients analyzed, the sellar complete type was the most prevalent pneumatization pattern, observed in 160 cases (63.2%). The sellar incomplete type occurred in 70 cases (27.7%), while the presellar type was identified in 22 cases (8.7%). SS agenesis was documented in one case (0.4%), and notably, the conchal type was absent (0%) in this cohort. No significant associations were identified between pneumatization type and sex or age. The distribution of main types by sex and age is summarized in Table 1.

### 3.2. Sphenoid Sinus Pneumatization—Extension Patterns

Anterior extension was present in 102 cases (20.2% of cases)—68 patients (26.8%), divided between bilateral (34 cases, 13.4%) and unilateral (34 cases, 13.4%) patterns.

Lateral extension was identified in 236 of 506 sides (46.6%). The most frequent subtype was the full-lateral (GW + PP) extension, detected in 58 sides (11.5%), followed by GW extension in 56 sides (11.1%) and combined full-lateral + LW extension in 48 sides (9.5%). Other lateral subtypes were less commonly observed.

Posterior (clival) extension was present in 161 cases (63.6%), with the subdorsal subtype being most prevalent (136 cases, 53.8%). Dorsal (6.3%), occipital (2.8%), and combined (0.8%) forms occurred less frequently.

No significant associations were found between any extension pattern and sex or laterality. The detailed distribution of anterior, posterior, and lateral extensions by sex and side is provided in Table 2.

### 3.3. Adjacent Neurovascular Structures

Regarding the ICA, a typical bilateral course was identified in 183 patients (72.3%). Bilateral ICA protrusion was observed in 40 patients (15.8%), while bilateral combined protrusion and dehiscence occurred in 4 patients (1.6%). Isolated unilateral dehiscence without protrusion was identified in only one patient. The distribution of ICA variants by side and sex is presented in Table 3.

For the ON, a typical bilateral course was observed in 185 patients (73.1%). Bilateral ON protrusion occurred in 24 patients (9.5%), and bilateral combined protrusion and dehiscence in 2 patients (0.8%). Detailed ON distribution by side and sex is shown in Table 3.

The VN demonstrated a bilateral typical course in 159 patients (62.8%). Bilateral VN protrusion was present in 21 patients (8.3%), and an additional 21 patients (8.3%) exhibited bilateral combined protrusion and dehiscence. The corresponding data are included in Table 3.

Regarding the maxillary nerve (MN/V2), a bilateral typical course was identified in 220 patients (87.0%). Bilateral MN protrusion occurred in 3 patients (1.2%), while no cases of bilateral combined protrusion and dehiscence were observed (0%). The distribution of MN variants is summarized in Table 3.

Onodi (sphenoethmoidal) cells were identified in 38 patients (15.0%), comprising 12 bilateral (4.7%) and 26 unilateral (10.3%) cases. Among these, pneumatization extending to the anterior clinoid process (LW extension pattern) was observed in 12 patients (9 unilateral and three bilateral), leading to ON exposure or protrusion within the Onodi cell in nearly all cases.

### 3.4. Association Between Sphenoid Sinus Pneumatization Types and Adjacent Neurovascular Structures

With respect to the main SSP types, the aplasia, presellar, and sellar incomplete subtypes were not associated with protrusion of any evaluated neurovascular structure. In contrast, the sellar complete type exhibited a tendency toward co-occurrence with ICA protrusion (with or without dehiscence) in 35.1% of cases, compared with aplasia (0%), presellar (6.8%), and sellar incomplete (0.7%). This association, however, was not statistically strong. Within the sellar complete group, the dorsal subtype demonstrated a significant association with ICA protrusion (81.2%) (Table 4). The OR analysis demonstrated two statistically significant results: the sellar complete pneumatization increases odds for ICA protrusion/dehiscence with an estimated OR of 5.28 (95% CI: 1.84–15.17, *p* = 0.002), and the sellar complete pneumatization was also associated with increased odds for ON protrusion/dehiscence with a calculated OR of 4.51 (95% CI: 1.36–15.01, *p* = 0.014).

In terms of lateral extension patterns, the LW extension was associated with protrusion of both the ICA and ON, with a particularly strong correlation for the ON (96%). VN protrusion was not evaluated for this pattern due to a lack of anatomical proximity. Conversely, both the full-lateral and PP extensions demonstrated notable associations with VN protrusion—79.9% and 56.1%, respectively (Table 4). The OR analysis demonstrated three statistically significant results: the PP extension had increased odds for VN protrusion/dehiscence with an OR of 8.08 (95% CI: 4.60–14.18, *p* < 0.001), the GW extension with the MN protrusion/dehiscence had an OR of 6.97 (95% CI: 3.19–15.20, *p* < 0.001), and the LW extension also showed a strong significant association with ON protrusion/dehiscence (*p* < 0.001).

Finally, the anterior extension type did not significantly affect the course or protrusion of any of the examined neurovascular structures (*p* > 0.05) (Table 4).

## 4. Discussion

In this retrospective analysis of 253 adult skull base CT scans, the sellar complete type was the most prevalent SSP pattern (63.2%), followed by the sellar incomplete (27.7%) and presellar (8.7%) types. At the same time, agenesis was rare (0.4%). Among directional extensions, posterior (63.6%) and lateral (46.6%) types predominated, whereas anterior extensions (26.8%) were less frequent. Significantly, posterior and lateral extensions were strongly associated with protrusion or dehiscence of the ICA, ON, and VN. Furthermore, Onodi (sphenoethmoidal) cells were identified in 15% of patients, most often in proximity to the ON and ICA, particularly when associated with LW extension. To the authors’ knowledge, this is the first radioanatomical study performed in the Greek population. Previously, only one study investigated the SS pneumatization patterns in cadavers performed by Greek researchers, but within a German population [21].

### 4.1. Anatomical Considerations of Sphenoid Sinus Pneumatization (SSP)

The SS demonstrates remarkable morphological variability, reflecting the interplay between embryologic development, postnatal aeration, and population-specific factors. Pneumatization begins in early childhood—usually visible radiographically by the first year of life—and continues into adolescence before reaching adult patterns [13,14]. Agenesis or hypoplasia of the sinus is uncommon, while well-pneumatized sellar variants represent the typical adult configuration [22].

Building upon these systems, Kiciński et al. [23] recently proposed a process-oriented classification, defining pneumatization into the LW (type I), GW (type II), and PP (type III), each further subdivided by vertical relation to key foramina—the optic canal (OC), foramen rotundum (FR), and Vidian canal (VC). This refined approach provides enhanced anatomical resolution, particularly relevant for pediatric skull bases and cases exhibiting asymmetrical development.

### 4.2. Anatomical Considerations of Neurovascular Relationships

Anatomically, the SS forms the central hub of the skull base and is intimately related to vital neurovascular structures, including the ICA, ON, VN, and the MN. The degree and direction of pneumatization directly influence the protrusion or dehiscence of these structures into the SS cavity, as well as the thickness of their osseous coverings. Prior studies consistently demonstrated that greater pneumatization correlates with higher frequencies of neurovascular protrusion and bony dehiscence [24,25,26]. In cadaveric studies, Cho et al. [24] reported ON bulging in 56%, ICA in 34%, MN in 41%, and VN in 52% of cases, with thinning or absence of bone most frequent in sellar complete sinuses. Refaat et al. [26] similarly found that postsellar pneumatization significantly increased the prevalence of ICA (50.6%), ON (52.3%), VN (56.9%), and MN (54.1%) protrusion or dehiscence. Stoković et al. [25] emphasized that sellar and postsellar types are independently associated with multiple simultaneous protrusions, irrespective of sinus size.

The relationships between the SS and FR or VC are less extensively studied. Mohebbi et al. [27] described variable proximity of these foramina to the SS walls, underlining the need for individualized preoperative mapping.

Our findings are consistent with these trends. Bilateral ICA protrusion was observed at 15.8%, ON protrusion in 9.5%, with combined protrusion and dehiscence in 1.6% and 0.8%, respectively. VN protrusion was identified in 8.3% and MN protrusion in 1.2% of cases. While these frequencies are lower than those reported in cadaveric studies, they align with large radiologic studies from Egyptian [28] and Southeast Asian [20] populations. Notably, PP, GW, LW, and dorsal clival extensions were strongly associated with ICA, ON, and VN protrusion/dehiscence, underscoring that pneumatization pattern, rather than population variation, is the primary determinant of neurovascular exposure risk.

Moreover, a key contribution of this study is the quantification of risk through OR analysis, providing a predictive framework for preoperative planning. While previous studies have noted the association between pneumatization and neurovascular protrusion, our data demonstrates that a sellar complete type increases the odds of ICA and ON exposure by 5.28 and 4.51 times, respectively. Lateral extensions were also significant predictors of neurovascular risk. The VN protrusion in the presence of PP extension (with an OR of 8.08) indicates that this is a critical anatomical variant, while the LW extension had a very strong association with the ON protrusion (96% of cases with this extension) suggesting that it should be considered as an almost definitive indicator of ON exposure.

### 4.3. Surgical Significance of Morphological Variations

The close anatomical relationship between the SS and critical neurovascular structures reinforces the necessity of meticulous preoperative imaging assessment in endoscopic and transsphenoidal skull base surgery. Variations in SS type, extension, and septation affect both the feasibility of surgical corridors and the risk profile for vascular or neural injury. In many cases, these factors directly influence approach selection, as outlined in recent skull base surgical literature. Well-pneumatized sellar and postsellar sinuses provide wide exposure of the sella and parasellar regions, facilitating safe and efficient dissection [29]. Conversely, presellar or conchal types require extensive drilling, navigation assistance, and angled visualization, increasing operative complexity and duration [30]. Our findings support this observation: 63.2% of cases demonstrated sellar complete pneumatization, providing optimal anatomy for transsphenoidal access, whereas agenesis (0.4%) and presellar variants (8.7%) were associated with restricted surgical corridors.

Posterior and lateral pneumatization, extending into the clivus, GW, LW, or PP, often results in neurovascular protrusion or dehiscence, heightening the risk of ICA injury, optic neuropathy, or VN damage [28]. Our data confirm that such extensions significantly increase the exposure of ICA, ON, and VN, in agreement with Egyptian, Southeast Asian, and European studies [20,25,28]. Specifically, the LW extension strongly correlated with OC and sphenoethmoidal cell variations, emphasizing the importance of vigilance during OC decompression or anterior clinoidectomy.

Onodi (sphenoethmoidal) cells, present in 15% of this cohort, represent a significant risk factor for injury to the ON and ICA due to their proximity to these structures. This prevalence aligns with prior reports (7–24%) [18]. Tawfik et al. [28] further demonstrated a significant association between Onodi cells and ON dehiscence, underscoring the critical value of preoperative CT identification.

The evolution from microscopic to endoscopic skull base surgery has expanded surgical boundaries beyond the sella to include the clivus, cavernous sinus, and planum sphenoidale [29]. While endoscopy enhances visualization, the SSP pattern defines the anatomical limits of safety. Our findings demonstrate that sellar complete and posterior extensions provide favorable access but simultaneously increase ICA and ON exposure. Notably, ON injury risk rises dramatically when ≥50% of its circumference is exposed within the sinus cavity [31]. Thus, surgical planning must balance the benefits of broader corridors against the heightened risk of neurovascular dehiscence [29]. El Kammash et al. [30] highlighted the impact of anatomy on surgical complications, reporting an intraoperative cerebrospinal fluid leak rate of 18.7% in patients with extended pneumatization. Our findings echo this trend- posterior and lateral recesses often expose critical neurovascular structures, underscoring the importance of comprehensive radiological mapping to anticipate technical challenges and minimize intraoperative morbidity.

The prospective utility of the current study lies in its transition from descriptive anatomy to a predictive model for skull base surgery. The quantification of the risk of neurovascular protrusion/dehiscence according to specific SS pneumatization patterns and extensions with statistically significant differences and ORs provides important insights for surgeons during the preoperative assessment. Identification of PP or LW extension can be a predictor for VN or ON exposure, respectively. This prediction and confirmation through preoperative CT scan allow for patient-specific customization of surgical corridors and approaches. Nevertheless, from an anatomical and research point of view, the current study provides the first comprehensive analysis of the SS in the Greek population—to the authors’ knowledge.

### 4.4. Strengths and Limitations

This study offers several strengths. First, it constitutes one of the most extensive radiologic analyses of SSP and neurovascular relationships in a European (Greek) adult cohort. Second, the combined use of sinus CT and CTA enabled precise correlation between osseous and vascular anatomy, thereby improving detection of protrusion and dehiscence. Third, the evaluation by a multidisciplinary panel (neurosurgeon, skull base otolaryngologist, and anatomist) ensured expert consensus and minimized bias. Finally, the study adopted standardized classification systems and assessed interrater reliability statistically, reinforcing methodological rigor.

However, several limitations must be acknowledged. The study’s retrospective nature and reliance solely on radiologic imaging preclude analysis of dynamic or intraoperative correlations. The single-center design limits generalizability beyond the Greek population. Although high-resolution CT and CTA enhance visualization, skinny bony partitions may have been underestimated, particularly in cases of partial dehiscence. The >50% canal exposure threshold for defining protrusion, though consistent with prior research, may not fully encompass clinical variability. Lastly, the absence of operative outcome data prevents direct assessment of how these anatomical patterns influence intraoperative safety or complications.

## 5. Conclusions

This study offers a comprehensive radiological characterization of SSP patterns and their neurovascular relationships within a Greek adult population. The predominance of the sellar complete type and the frequent presence of posterior and lateral extensions align with international data. Sellar complete pneumatization and lateral extensions into the PP and GW significantly increase the odds of ICA, ON, and VN protrusion/dehiscence. Thus, a meticulous preoperative radiological assessment of SS anatomy remains important for skull base surgeons, facilitating secure surgical planning, optimal exposure, and minimizing operative complications in endoscopic transsphenoidal and extended skull base procedures.

## Figures and Tables

**Table 1:** Descriptive statistics of the main types of sphenoid sinus pneumatization (SSP) according to sex and age. Data are presented as frequency (percentage) and mean age ± standard deviation. The single case of sphenoid sinus agenesis was excluded from the statistical analysis due to a deviation from the test assumptions.

SS Main Types	Total (*n* = 253)	Females (*n* = 124)	Males (*n* = 129)	*p*-Value	Age Distribution	*p*-Value
**Sellar Complete**	160 (63.2%)	81 (65.3%)	79 (61.2%)	0.288	50.46 (14.6)	0.634
**Sellar Incomplete**	70 (27.7%)	33 (26.6%)	37 (28.7%)		53.63 (15.8)	
**Presellar**	22 (8.7%)	9 (7.3%)	13 (10.1%)		54.14 (14.8)	
**Agenesis ^†^**	1 (0.4%)	1 (0.8%)	0 (0%)		70.00 (-)	

*p* < 0.05 is considered statistically significant. ^†^ Single case excluded from inferential analysis. The sign (-) represents the absence of standard deviation (SD) on this type because it is only one patient.

**Table 2:** Descriptive statistics of sphenoid sinus pneumatization (SSP) extension patterns according to side and sex. Data are presented as the number of cases (frequency). * Asterisk indicates statistically significant results.

SSP Extension Patterns	Total (*n* = 506)	Left (*n* = 253)	Right (*n* = 253)	*p*-Value	Females (*n* = 248)	Males (*n* = 258)	*p*-Value
**Anterior**	102 (20.2%)	52 (20.6%)	50 (19.8%)	0.825	42 (16.9%)	60 (23.3%)	0.076
**Lateral**	236 (46.6%)	121 (47.8%)	115 (45.5%)	0.366	117 (47.2%)	119 (46.1%)	0.184
**LW**	35 (6.9%)	13 (5.1%)	22 (8.7%)		11 (4.4%)	24 (9.3%)	
**GW**	56 (11.1%)	34 (13.4%)	22 (8.7%)		31 (12.5%)	25 (9.7%)	
**PP**	23 (4.5%)	10 (4%)	13 (5.1%)		14 (5.6%)	9 (3.5%)	
**FL**	58 (11.5%)	29 (11.5%)	29 (11.5%)		33 (13.3%)	25 (9.7%)	
**LW + GW**	11 (2.2%)	5 (2%)	6 (2.4%)		6 (2.4%)	5 (1.9%)	
**LW + PP**	5 (1%)	4 (1.6%)	1 (0.4%)		3 (1.2%)	2 (0.8%)	
**LW + FL**	48 (9.5%)	26 (10.3%)	22 (8.7%)		19 (7.7%)	29 (11.2%)	
**Posterior**	161 (63.6%)	-	-	-	81 (65.3%)	80 (62%)	<0.001 *
**Dorsal**	16 (6.2%)				3 (2.4%)	13 (10.1%)	
**Subdorsal**	136 (53.8%)				73 (58.9%)	63 (48.8%)	
**Occipital**	7 (2.8%)				5 (4%)	2 (1.6%)	
**Combination**	2 (0.8%)				0 (0%)	2 (1.6%)	

**Notes**: Data are presented as the number of cases (percentage). Asterisk indicates a statistically significant result at *p* < 0.05.* Anterior and lateral extensions were evaluated bilaterally (*n* = 506 sides), whereas posterior extensions were assessed per case (*n* = 253). Abbreviations: SSP, sphenoid sinus pneumatization; LW, lesser wing; GW, greater wing; PP, pterygoid process; FL, full lateral.

**Table 3:** Descriptive statistics of the course of adjacent neurovascular structures (ICA, ON, VN, MN) according to side and sex. Data are presented as the number of cases (frequency). *ICA: internal carotid artery, ON: optic nerve, VN: Vidian nerve, MN: Maxillary nerve, Pt: protrusion, Dh: Dehiscence*.

Parameters	Total (*n* = 506)	Left (*n* = 253)	Right (*n* = 253)	*p*-Value	Females (*n* = 248)	Males (*n* = 258)	*p*-Value
**ICA Pt**	103 (20.4%)	52 (20.6%)	51 (20.2%)	0.728	42 (16.9%)	61 (23.6%)	0.139
**ICA Pt + Dh**	14 (2.8%)	8 (3.2%)	6 (2.4%)		9 (3.6%)	5 (1.9%)	
**ON Pt**	83 (16.4%)	40 (15.8%)	43 (17%)	0.335	31 (12.5%)	52 (20.2%)	0.051
**ON Pt + Dh**	15 (3%)	5 (2%)	10 (4%)		8 (3.2%)	7 (2.7%)	
**VN Pt**	78 (15.4%)	40 (15.8%)	38 (15%)	0.242	44 (16.9%)	34 (13.2%)	0.232
**VN Pt + Dh**	64 (12.6%)	38 (15%)	26 (10.3%)		27 (10.9%)	37 (14.3%)	
**MN Pt**	28 (5.5%)	17 (6.7%)	11 (4.3%)	0.320	14 (5.6%)	14 (5.4%)	0.278
**MN Pt + Dh**	11 (2.2%)	7 (2.8%)	4 (1.6%)		8 (3.2%)	3 (1.2%)	

**Table 4:** Comparison between the variation—typical or protrusion/dehiscence—of each neurovascular structure (ICA, ON, VN, V2) and the SS pneumatization pattern. All the variations are presented as cross tabulation with the SS pneumatization pattern and its extensions. The results provide statistically significant association between the presence of protrusion/dehiscence of the neurovascular structures with specific patterns, and they are highlighted in red color. Data are presented as number of cases (frequency). *ICA: Internal Carotid Artery, ON: Optic Nerve, VN: Vidian nerve, MN: Maxillary nerve, SI: Sellar Incomplete, SC: Sellar Complete, FL: Full Lateral, GW: Greater Wing, PP: Pterygoid Process, LW: Lesser Wing, Pt: protrusion, Dh: Dehiscence, ND: Not Detected,* * Asterisk indicates statistically significant results.

Pneumatization Types		ICA		ON		VN		MN		*p*-Value
		Typical (*n* = 389)	Pt/Pt + Dh (*n* = 117)	Typical (*n* = 408)	Pt/Pt + Dh (*n* = 98)	Typical (*n* = 364)	Pt/Pt + Dh (*n* = 142)	Typical (*n* = 467)	Pt/Pt + Dh (*n* = 39)	
**Main**	**Aplasia**	2 (100%)	0 (0%)	2 (100%)	0 (0%)	2 (100%)	0 (0%)	2 (100%)	0 (0%)	<0.001 *
	**Conchal**	ND	ND	ND	ND	ND	ND	ND	ND	
	**Presellar**	41 (93.2%)	3 (6.8%)	41 (93.2%)	3 (6.8%)	44 (100%)	0 (0%)	44 (100%)	0 (0%)	
	**SI**	137 (99.3%)	1 (0.7%)	129 (93.5%)	9 (6.5%)	127 (92%)	11 (8%)	136 (98.6%)	2 (1.4%)	
	**SC**	209 (64.9%)	113 (35.1%)	236 (73.3%)	86 (26.7%)	191 (59.4%)	141 (40.6%)	285 (88.5%)	37 (11.5%)	
**Posterior Extension**	**Subdorsal**	192 (70.6%)	80 (29.4%)	208 (76.5%)	64 (23.5%)	171 (62.9%)	101 (37.1%)	246 (90.4%)	26 (9.6%)	<0.001 *
	**Dorsal**	6 (18.8%)	26 (81.2%)	18 (56.3%)	14 (43.7%)	11 (34.4%)	21 (65.6%)	23 (71.9%)	9 (28.1%)	
	**Occipital**	9 (64.3%)	5 (35.7%)	9 (64.3%)	5 (35.7%)	7 (50%)	7 (50%)	12 (85.7%)	2 (14.3%)	
	**Combo**	2 (50%)	2 (50%)	1 (25%)	3 (75%)	2 (50%)	2 (50%)	4 (100%)	0 (0%)	
**Lateral Extension**	**FL**	49 (46.2%)	57 (53.8%)	58 (54.7%)	48 (45.3%)	16 (15.1%)	90 (79.9%)	75 (70.8%)	31 (29.2%)	<0.001 *
	**GW**	50 (74.6%)	17 (25.4%)	56 (83.6%)	11 (16.4%)	35 (52.2%)	32 (47.8%)	59 (88.1%)	8 (11.9%)	
	**PP**	18 (64.3%)	10 (35.7%)	23 (100%)	0 (0%)	12 (42.9%)	16 (56.1%)	28 (100%)	0 (0%)	
	**LW**	43 (43.4%)	56 (56.6%)	4 (4%)	95 (96%)	44 (44.4%)	45 (55.6%)	81 (81.8%)	18 (18.2%)	
**Anterior Extension**	**Yes**	87 (85.3%)	15 (14.7%)	91 (89.2%)	11 (10.8%)	73 (71.6%)	29 (28.4%)	94 (92.2%)	8 (7.8%)	>0.05

## Data Availability

The original contributions presented in this study are included in the article. Further inquiries can be directed to the corresponding author.
